# P49 Belimumab as a potential therapy for lupus enteritis – a case report

**DOI:** 10.1093/rap/rkae117.080

**Published:** 2024-11-05

**Authors:** Sahil Jain, Abdulrahman Khormi, Louise Nel, Shirish Sangle, David D’Cruz

**Affiliations:** Louise Coote Lupus Unit, Guy’s Hospital, London, United Kingdom; Prince Sattam University Medical College, AlKharj, Saudi Arabia; Louise Coote Lupus Unit, Guy’s Hospital, London, United Kingdom; Louise Coote Lupus Unit, Guy’s Hospital, London, United Kingdom; Louise Coote Lupus Unit, Guy’s Hospital, London, United Kingdom

## Abstract

**Introduction:**

Systemic lupus erythematosus (SLE) is a multisystem autoimmune disease with varied clinical manifestations. Gastrointestinal (GI) complaints are common in 40–60% of SLE patients. Most are mild; however severe life-threatening manifestations may occur. Lupus enteritis (LE) is a rare but severe GI manifestation of SLE, characterised by vasculitis or inflammation of the bowel. Belimumab is a recombinant human monoclonal antibody against the soluble B-lymphocyte stimulator protein (BLyS) and was the first licensed treatment for SLE and lupus nephritis. Herein, we report a case of lupus enteritis that responded well to belimumab therapy.

**Case description:**

We present a 42-year-old Sri Lankan woman who was diagnosed with SLE in 2015 based on arthritis, Raynaud’s, haemoproteinuria, positive antinuclear antibodies (ANA), anti-double stranded DNA (dsDNA), and anti-Ro antibodies with low complements C3, C4. Renal biopsy confirmed class III a+c lupus nephritis. She was managed with prednisolone, mycophenolate mofetil, hydroxychloroquine and ramipril. She responded well to treatment.

She was admitted in August 2018 with recurrent abdominal pains, distension, diarrhoea, nausea, vomiting, 20kg unintentional weight loss, arthritis, lymphadenopathy, and peripheral oedema. Over the last 12 months, she had 5 admissions with similar GI symptoms, which were managed as viral gastroenteritis.

Investigations showed nephrotic range proteinuria - urine PCR 330 (0-15 mg/mmol), low albumin = 27 (40-52 g/L), positive anti-dsDNA antibodies = 211 (0-10 IU/ml), CRP 7 (0-4 mg/L), ESR 44 (0-12 mm/hr) with low complements C3 0.36 (0.90-1.80 g/L) and C4 0.07 (0.10-0.40 g/L). Contrast enhanced computed tomography (CECT) scan of the abdomen revealed diffuse oedema of the large bowel extending from the splenic flexure to the rectum, several areas of small bowel thickening, peritoneal enhancement, and moderate volume ascites. Infective causes for colitis were ruled out including abdominal tuberculosis. Sigmoidoscopy and biopsy ruled out inflammatory bowel disease, whereas surgical and gastroenterology reviews ruled out other causes for her presentation.

She was diagnosed with lupus enteritis with flare of lupus nephritis and managed with pulsed intravenous methylprednisolone followed by tapering high dose oral prednisolone. Her symptoms gradually improved, and she was discharged. Unfortunately, over the next month, she had recurrent admissions with flares of lupus enteritis requiring further intravenous methylprednisolone. As a result, she was commenced on monthly IV belimumab.

Since starting belimumab, she has not had any further flares of lupus enteritis and her anti-dsDNA antibody and complements C3, C4 levels have normalised.

**Discussion:**

Lupus enteritis (LE) presents with non-specific GI symptoms such as abdominal pain (most common), nausea, vomiting, diarrhoea and ascites. In most cases, it develops months to years after SLE diagnosis but in some cases, may be the presenting feature. Its pathogenesis remains unknown; however, mesenteric vasculitis and/or thromboses are thought to play a role.

Contrast enhanced computed tomography (CECT) abdomen is the gold-standard investigation and usually shows bowel wall oedema, dilated bowel loops (>3 cm), bowel wall thickening with peripheral enhancement (“target sign”/”double halo sign”), engorgement of mesenteric vessels with a palisade pattern (“comb-sign”), increased attenuation of mesenteric fat and/or ascites. Laboratory investigations usually reveal markers of active lupus. Significant proteinuria (>0.5 g/24 h) is usually found in around 50% of patients.

LE is very steroid responsive, with most patients achieving disease remission with steroids alone. Additional immunosuppression (such as cyclophosphamide, MMF, azathioprine, HCQ or rituximab) may be used concomitantly, for other manifestations of SLE or in those with relapsing or refractory disease. Belimumab has previously been licensed for use in SLE and lupus nephritis; however, there is sparse literature about its use in lupus enteritis. Our patient responded very well to belimumab, with both clinical and immunological remission.

**Key learning points:**

• Lupus enteritis is a rare but severe gastrointestinal (GI) manifestation of SLE that presents with non-specific GI symptoms such as abdominal pain (most common), nausea, vomiting, diarrhoea and ascites. Clinicians managing SLE patients should have a high index of suspicion regarding the diagnosis, else it may be easily missed leading to recurrent admissions and delayed treatment.

• Contrast enhanced CT abdomen is the gold-standard investigation and shows characteristic changes - bowel wall oedema, dilated bowel loops (>3 cm), bowel wall thickening with peripheral enhancement (“target sign”/”double halo sign”), engorgement of mesenteric vessels with a palisade pattern (“comb-sign”), increased attenuation of mesenteric fat and/or ascites

• Lupus enteritis is very steroid responsive, with most patients achieving disease remission with steroids alone. Some patients with other SLE manifestations, or relapsing/refractory disease may require additional immunosuppression. Belimumab may be another option to consider in such patients.

